# Face-to-face or face-to-screen? Undergraduates' opinions and test performance in classroom vs. online learning

**DOI:** 10.3389/fpsyg.2014.01278

**Published:** 2014-11-12

**Authors:** Nenagh Kemp, Rachel Grieve

**Affiliations:** Discipline of Psychology, School of Medicine, University of TasmaniaHobart, TAS, Australia

**Keywords:** online learning, e-learning, face-to-face learning, university, discussion forums

## Abstract

As electronic communication becomes increasingly common, and as students juggle study, work, and family life, many universities are offering their students more flexible learning opportunities. Classes once delivered face-to-face are often replaced by online activities and discussions. However, there is little research comparing students' experience and learning in these two modalities. The aim of this study was to compare undergraduates' preference for, and academic performance on, class material and assessment presented online vs. in traditional classrooms. Psychology students (*N* = 67) at an Australian university completed written exercises, a class discussion, and a written test on two academic topics. The activities for one topic were conducted face-to-face, and the other online, with topics counterbalanced across two groups. The results showed that students preferred to complete activities face-to-face rather than online, but there was no significant difference in their test performance in the two modalities. In their written responses, students expressed a strong preference for class discussions to be conducted face-to-face, reporting that they felt more engaged, and received more immediate feedback, than in online discussion. A follow-up study with a separate group (*N* = 37) confirmed that although students appreciated the convenience of completing written activities online in their own time, they also strongly preferred to discuss course content with peers in the classroom rather than online. It is concluded that online and face-to-face activities can lead to similar levels of academic performance, but that students would rather do written activities online but engage in discussion in person. Course developers could aim to structure classes so that students can benefit from both the flexibility of online learning, and the greater engagement experienced in face-to-face discussion.

## Introduction

With changing student lifestyles and fast-developing technology, universities are increasingly offering more “flexible” learning environments. Commensurate with the opportunities that technological advances afford, for over a decade (Imel, 2002) the provision of online, e-learning experiences has undergone rapid expansion in the higher education sector. Today, online learning is part of the student experience for a substantial proportion of university students in a variety of countries (e.g., Ituma, 2011; Otter et al., 2013; Tucker et al., 2013). The current study aimed to compare students' experience and performance in both online and traditional face-to-face learning experiences.

The rise of e-learning has helped to encourage students to take on more responsibility for their own acquisition of knowledge (Ituma, 2011). In a traditional, teacher-centered model of teaching, the lecturer transmits knowledge to students, with little input from those students (Harden and Crosby, 2000; Prosser et al., 2005). However, the shift to less traditional classes has coincided with a greater focus on more student-centered learning, with the lecturer facilitating or managing the students' learning, rather than simply transmitting information (Balluerka et al., 2008). Because of the more self-directed learning assumed to occur in online environments, online learning may have the potential to produce more in-depth discussions and to improve the quality of learning, as well as having the practical benefits of encouraging wider student participation and increasing the cost-effectiveness of education, compared to traditional face-to-face learning (Smith and Hardaker, 2000; Alexander, 2001). A timely example is that of flipped classrooms, whereby the students engage in active learning (often via vodcasts or in online discussions) and the instructor provides support and scaffolding (Strayer, 2012).

Given the potential pedagogical advantages of online learning, there is a pressing need to formulate an evidence-based understanding of best practice in this area. However, despite the increasing research interest in e-learning, there seems to be little consistency in the training that lecturers receive in developing online materials. This may be an artifact of a diverse research base. For example, many studies have focused on the efficiency, content and delivery method that teaching staff have developed (e.g., Rossman, 1999; Twigg, 2003; O'Neill et al., 2004), while the perceptions and experiences of the students themselves have been largely neglected (Alexander, 2001; Holley and Oliver, 2010; Ituma, 2011). Some teaching staff seem to perceive web-based platforms simply as an alternative method for presenting the traditional content, whereas others might look for more innovative ways of using such platforms to improve students' engagement and thus their learning outcomes (Holley and Oliver, 2010; Ituma, 2011). Much of the initial experience of e-learning failed to live up to learners' expectations (Imel, 2002), partly because of technological constraints, and partly because of the early instructional approaches taken (Anderson and Dron, 2011). Even today, students in different courses can have quite different experiences of online learning. There is a clear need for more research into what does and does not work in online learning, but also for a focus on the student experience in the increasingly digital landscape of tertiary education.

The worldwide trend toward online learning provision has resulted in numerous online-only courses, and universities in Australia are no exception (e.g., Bell et al., 2002; Tucker et al., 2013). However, the student experience in online classes is a different one from in traditional face-to-face classes, and patterns of engagement seem to differ between the two (Robinson and Hullinger, 2008). For example, Otter et al. (2013) found that students in online-only classes felt more disconnected from their peers and lecturers, more obliged to be self-directed in their studies, and less aided by their lecturer, than their lecturers believe them to be. Students can also feel daunted by the technological expectations of online study, especially if they start off without sufficient technical knowledge or support (Zhang and Perris, 2004; Holley and Oliver, 2010).

Most of the research to date has focused on courses offered entirely online. Yet, an increasing number of face-to-face courses are beginning to incorporate some online components, in which traditional in-class activities are supplemented, rather than replaced, with online activities (Ituma, 2011). However, very little is known how students respond to this kind of “blended e-learning,” especially when they have enrolled in a face-to-face class, rather than one which they expect in advance to be completed online. This represents a substantial gap in the educational literature, as there are potentially important implications for student engagement, performance, and attrition. In general, student engagement in traditional classes is positively associated with student engagement and academic performance, although the magnitude of these effects may be small (e.g., Carini et al., 2006). Some research suggests that participation in learning technology can itself increase engagement and learning (Chen et al., 2010), and flipped classrooms are emerging as a promising student-centered paradigm (e.g., Galway et al., 2014). However, a deeper consideration of these positive outcomes is warranted, as outcomes seem to depend on the nature of the online activities, and the way that students interact with them. For example, Davies and Graff (2005) found that students who interacted and participated more in online discussion did not show significantly better academic performance than students who were less involved in that discussion. In contrast, Evans et al. (2004) showed that students performed much better when their online course material was accessible via an interactive, navigable format than via a series of scrollable web-pages. Thus, there is mixed evidence about the extent to which some online activities might help or hinder students' marks, compared to more traditional, face-to-face classes.

There are various reasons to expect that students might prefer at least some aspects of online learning to traditional classes. Previous researchers have suggested that in contrast to the faster, real-time pace of face-to-face classes, the extra time available for online activities might allow students to think about course material more critically and reflectively, leading to deeper understanding of the course content (e.g., Ramsden, 1992; Robinson and Hullinger, 2008). Others have suggested that the less confrontational or personal nature of e-learning might encourage shyer students to engage more, or to feel less pressure than in face-to-face interactions (Warschauer, 1997; Hobbs, 2002).

However, there are also reasons for which students might prefer more traditional, in-class activities. Although social connectedness can be derived online (Grieve et al., 2013), most students feel that face-to-face contact is essential for building a sense of community (Conole et al., 2008). Even when classes are only partially online, students may feel that online discussion detracts from this feeling of community with their peers and tutor. Further, at a practical level, students need to exercise more self-motivation to complete activities online, compared to in-class, where that role of motivator is taken on by the lecturer (Upton, 2006). Thus, it is important to explore students' perceptions of both online and face-to-face learning experiences, rather than just one or the other.

At our university, there is currently a focus on incorporating more online material into face-to-face units, by replacing some weekly practical classes with self-directed, online activities. The primary motivation is a financial one, as online classes do not require paid tutors, and lecturing staff are not paid any more for developing online material than in-class material. A secondary motivation is the general assumption that students prefer the flexibility and opportunity for self-directed learning provided by online activities. However, there does not seem to have been any assessment of students' academic outcomes, or their overall experience, in terms of online learning within this context.

The current research was therefore designed to examine the performance and perceptions, in both face-to-face and online learning experiences, of Australian undergraduate students who were enrolled in traditional face-to-face units which incorporated some online components. We took a two-fold approach, with the aim of providing an integrated picture of both objective and subjective outcomes. To this end, we compared both students' academic performance and their qualitative comments on their learning experience, between offline and online modalities, as described below.

In order to control for individual differences and thus increase the statistical power of the study, we tested the same students on both offline and online tasks, rather than having separate groups for each modality. Alongside the empirical rigor of this within-groups design, we wished to maintain the authenticity of the measures, and thus chose to embed the tasks within the students' actual class learning experience, rather than in an artificial, lab-based study. We also wanted to ensure that instead of just observing students' behavior (e.g., counting their discussion contributions), we assessed both objective and subjective measures of their learning. The specific research questions were thus as follows:

After having engaged with course content either face-to-face or online, would students differ in:

1. Their academic marks on a short test of the concepts just learned?
2. Their preference ratings for online and face-to-face modalities?
3. The themes emerging in their justifications of these ratings?

## Study 1

In the first study, undergraduate psychology studied two carefully matched topics, one in a face-to-face class and the other online, with the order counterbalanced between two groups. We compared marks on the two topics, and examined the students' comments on their experience of completing the activities, one in-class and one online.

### Methods

#### Participants

Participants were 67 third-year (advanced level) undergraduate students at an Australian university, 13 male and 54 female. Their mean age was 24 years (*SD* 7.1 years), and all spoke English as a first language. The students took part in the study as part of their unit on developmental psychology, but gave consent for their data also to be used for research purposes. All were familiar and confident with the web-based platform used for delivering the online activities. The study received approval from the university's Human Research Ethics Committee.

#### Materials and Procedure

Participants completed written exercises, a class discussion, and a written test (worth 5% of their overall mark) on two academic topics, one week apart. The first topic concerned children's stages of cognitive development, and the second, children's stages of drawing development. Three classes of students participated. The classes were allocated to two groups (as described in the next paragraph), with the larger class allocated to Group 1 (*n* = 27), and the two smaller classes to Group 2 (*n* = 40). This meant that the two groups differed in size. However, there were no significant differences between the two groups in terms of age [*M* = 23.2 years (*SD* 6.2) and *M* = 26.0 years (*SD* 8.2), respectively], sex distribution (80 and 82% female), or overall performance in this unit [*M* = 62.4% (*SD* 11.6%) and *M* = 65.0% (*SD* 10.0%)], all *p*s > 0.1.

In Week 1, both groups of participants completed the face-to-face activities in their weekly practical class. Group 1 students discussed their recent experience of observing a child completing several tasks of conservation of matter (e.g., conservation of liquid, conservation of number, Piaget, 1954). The previous week, each student had individually administered these tasks to a 5- to 12-year-old child of their acquaintance. The few students who did not know a child to visit had viewed multiple online videos of children being administered these tasks and chosen the one on which they would like to answer the test questions. There were no differences in performance between these two sets of students. Group 2 students were given a series of self-portraits drawn by children aged 2–16 years, and a copy of Lowenfeld's (1939) stages of drawing development.

In class, the tutor initiated the discussion with more general questions, for example (for conservation, Group 1): “What did you find most difficult about giving the task, and how did you overcome this difficulty?,” and (for drawing, Group 2) “Do you think it would be easy or difficult to persuade a child of this age to produce a self-portrait? Do you think it would be easier with older or younger children?.” The questions later narrowed in on theory, with the tutor asking the students in Group 1 to discuss which cognitive stage they thought the child was in (in terms of Piaget's stage model of cognitive development, e.g., pre-operations or concrete operations), and how the wording of the adult's instructions could influence the child's performance. In Group 2, the tutor asked the students to decide which developmental stage each self-portrait seemed to represent (e.g., pre-schematic drawing, schematic drawing), and why. They also talked about the motor skills required for drawing. For both groups, discussions took place first in small groups and then as a whole class, over a 1-h period, with the questions provided by the tutor but the discussion structured by the students themselves. At the end of the class, both groups were then given a half-hour, in-class, written test on the topic that they had just studied (i.e., development of conservation or drawing):

1. Participants answered six short-answer questions structured as similarly as possible for the two topics (e.g., *Conservation*: Based on your observation, which Piagetian stage do you think this child is in, and why? *Drawing:* Based on the drawing, which of Lowenfeld's stages do you think this child is in, and why?)
2. Participants rated their preference for completing these activities face-to-face vs. online, on a five-point scale ranging from 1 (“much prefer to do in class”) to 5 (“much prefer to do online”), and were asked to identify “one thing that was good about doing the activities in class, not online,” and “one thing that would have been good about doing the activities online, not in class.”

During the following week (Week 2), both groups of participants completed the online activities at a time of their own choosing, for the topic that they had not covered in the previous week. Group 1 scrolled through the series of children's self-portraits, presented in PDF form, and were provided with an online copy of Lowenfeld's (1939) stages of drawing development. Group 2 were asked to think about the conservation tasks they had observed.

Both groups were asked to consider the same questions that had been considered in the face-to-face classes as described above, for drawing development (Group 1) and for cognitive development (Group 2). Students were then instructed to go to the online discussion board. They did not have to transcribe their answers for all of the questions that they had considered, but were asked to contribute their answers to the questions that they “had found most interesting,” or to explain any questions that they “had found particularly difficult, and why.” Students were also asked to read others' contributions, and to respond to them, “linking their experiences and answers with yours if you can.”

The opportunity to reflect on the materials and questions, and to contribute to the online discussion, was available for 5 days, with each participant contributing at least once. Each participant contributed to the discussion, as required (with at least one discussion post that covered several questions). The day after the online discussion board was closed, the online version of the test was made available, with the same questions as in the face-to-face version:

1. Participants responded in writing to the same six short-answer questions answered in class the week before.
2. Participants rated their preference for completing these activities face-to-face or online, and were asked to identify “one thing that was good about doing the activities online, not in class,” and “one thing that would have been good about doing the activities in class, not online.”

Students had 4 days in which to do the test, which was open for 30 min once it was begun. It is possible that the students completing the test online could have accessed extra information (from the internet) than the students completing the test in class (without internet access). However, trying to find further information during the limited time available for writing the test answers would not have been very helpful. The questions were focused on discussing the conservation or drawing performance of “their” child, and all students (in class and online) had access to a copy of Lowenfeld's stages, and all had been aware that they would need to revise the basics of Piaget's cognitive stage theory for the test.

In summary, both groups completed the same activities, discussion and tests, but in Week 1 both groups did so in face-to-face classes (on cognitive development for Group 1 and on drawing development for Group 2) and in Week 2 both groups did so online (on drawing development for Group 2 and on cognitive development for Group 1).

### Results and discussion

#### Class marks and preferences

Participants' marks on the two tests, as well as their stated preferences for studying each topic face-to-face or online, were calculated. Table 1 shows the means and standard deviations on each of these factors, for participants in the two groups. As seen in the table, students showed an overall preference for studying topics face-to-face rather than online, with their average preferences of around 2 hovering much closer to the “much prefer to do in class” end of the 5-point scale than the “much prefer to do online” end of the scale.

**Table 1:** **Study 1: Mean Marks and Modality Preferences across Task and Modality**.

	**Class modality**	**Cognition**	***n***	**Drawing**	***n***	**Overall**	***n***
Test mark (%)	Face-to-face	13.07 (2.72)	27	14.51 (2.25)	39	13.92 (2.53)	66
	Online	14.03 (2.20)	40	13.19 (2.60)	27	13.69 (2.39)	67
	Overall	13.64 (2.45)	67	13.97 (2.47)	66		
Modality pref. (1–5)	Face-to-face	1.96 (0.78)	27	2.22 (1.28)	41	2.15 (1.07)	67
	Online	2.25 (1.24)	40	1.77 (0.65)	26	2.06 (1.07)	66
	Overall	2.13 (1.07)	67	2.04 (1.09)	66		

For modality preference, 1 = much prefer to do in class (face-to-face), 5 = much prefer to do online.

A series of repeated-measures analysis of variance (ANOVAs) showed that there were no significant differences between the groups on any of the comparisons made. The two groups did not differ significantly in the marks they gained, in terms of either the topic they were tested on [*F*_(1, 65)_ = 0.67, *p* = 0.42, partial η^2^ = 0.01] or modality in which this test was presented [*F*_(1, 65)_ = 0.31, *p* = 0.58, partial η^2^ = 0.01]. Nor did they differ significantly in their modality preference, in terms of either the topic about which they were asked [*F*_(1, 65)_ = 0.42, *p* = 0.52, partial η^2^ = 0.01] or the modality in which they were asked it [*F*_(1, 65)_ = 0.05, *p* = 0.82, partial η^2^ = 0.001]. We followed up this significant group difference in modality preference by also considering whether there was a significant difference in terms of the *number* of students who (much) preferred to do the tasks in class and those who (much) preferred to do them online/did not mind either way. A chi-squared analysis confirmed that significantly more students preferred to complete the tasks in class (*n* = 47) than online/didn't mind (*n* = 20), χ^2^_(1)_ = 10.89, *p* < 0.001.

Pearson correlations were calculated to see if there was any association between participants' academic performance and their preference for learning face-to-face vs. online. We first considered participants' scores on the two topic-specific tests and their reported modality preference for each topic. The correlations were not significant for either the topic done online (*r* =−0.02, *p* = 0.85), or the topic done in class (*r* = 0.01, *p* = 0.95). This suggests that students did not simply prefer a modality because they felt they had performed better on the task in that modality, and nor did they perform better in the modality that they already preferred. We then looked at participants' final mark on this entire academic unit (*M* = 64.0%, *SD* = 10.7%), and calculated the correlation between this mark and their modality preference averaged over the face-to-face and online tasks (*M* = 2.09, *SD* = 0.92). The correlation was not significant (*r* = −0.11, *p* = 0.40), suggesting no consistent relationship between overall academic performance and preference for learning online or in class. Finally, we confirmed that there was no significant correlation between participant age and modality preference (*r* = 0.01, *p* = 0.97), nor between age and overall marks (*r* = 0.05, *p* = 0.69).

#### Qualitative comments

We also asked participants to note one thing that they liked better about completing the activities (exercises, discussion, and test) face-to-face, and one thing about what they liked better about completing them online. Participants provided their own freeform answers, which we then subjected to thematic analysis (Braun and Clarke, 2006). We chose thematic analysis rather than a more specific approach such as Interpretive Phenomenological Analysis (IPA) or Discourse Analysis, as we wished to identify repeated patterns of meaning, or themes, in the responses, but without attaching our analysis to any pre-existing theoretical framework. We followed Braun and Clarke's (2006) six phases to identify the themes as they emerged from an analysis of students' responses, rather than pre-existing themes being imposed on a grouping of the responses. Specifically, we (1) familiarized ourselves with the scope and nature of the students' responses to both the “in-class” and “online” questions, and (2) generated initial codes on the basis of a subset of 25 participants' responses, by identifying the overall point made in each response with a 2- to 4-word summary code, which we (3) collated into tentative themes and modified as we reviewed another 25 responses. These potential themes were then (4) reviewed to check how they fitted with the entire data set, and (5) the themes were refined into an exhaustive set of final themes for all responses, which were named and clearly defined. Finally, (6) the most representative example for each theme was selected, and the final analysis related back to the research questions. Although participants had been asked to provide only one reason for liking something about each modality, many provided two or even more reasons. We included the first one or two reasons given in our thematic analysis. Table 2 shows the proportion of responses that were categorized into each them, for face-to-face and online learning, for the whole sample, *n* = 67.

**Table 2:** **Study 1: Proportion of Responses per Theme, for Face-to-Face and Online Learning**.

**FACE-TO-FACE**							
**Theme**	**More engagement**	**Immediate feedback**	**No wish to read comments**	**Deeper understanding**	**Better flow of argument**	**Easier to review material**	**Other**
Reason 1 (*n* = 63)	0.56	0.24	0.08	0.06	0.02	0.02	0.02
Reason 2 (*n* = 39)	0.21	0.16	0.16	0.08	0.24	0.11	0.07
**ONLINE**							
**Theme**	**More convenient**	**Wider contributions**	**More detailed responses**	**More time to think**	**Faster to type**	**Less judgment**	**Other**
Reason 1(*n* = 62)	0.47	0.26	0.11	0.08	0.05	0.02	0.02
Reason 2 (*n* = 16)	0.06	0.25	0.38	0.13	0.06	0.06	0.06

In justifying their modality preferences about completing the activities face-to-face, the 67 participants provided 102 reasons overall. The themes that emerged were as follows:

- *More engagement:* This was the theme with the most responses, with students noting that they felt more engaged when the activities were completed in the social environment of a classroom setting, rather than online. Most also commented that they felt this greater engagement led to more meaningful discussion, for example, *I think that discussion face to face really allows you to think more deeply and bounce ideas of other people. Writing it online, felt like your answers had to be more formal and exact, whereas in class discussion I felt you could really bounce more possible ideas off each other before coming to a conclusion.*
- *Immediate feedback:* The next most common theme was that participants appreciated the fact that each comment they made in class immediately elicited a related comment from a peer, or a clarification from the tutor in real time, rather than having to wait hours for a response to their particular comment online. An illustrative example is, *You are able to directly discuss with tutor and peers and therefore directly receive feedback for your questions and others questions.*

Overall, 80% of the first reasons (and 37% of second reasons) that participants gave to illustrate the benefits of in-class activities fitted these themes of “more engagement” and “immediate feedback.” Some of remaining responses referred to more practical considerations:

- *No wish to read comments:* Some students noted that they had no wish to read the comments of their classmates online, although they were happy to listen to and interact with their classmates in real life.
- *Easier to review paper documents:* Others noted it was easier to review material in class, because they could spread out the pages in front of them on a table rather than scrolling through on screen. The other remaining responses fitted the more pedagogically motivated themes that in-class interaction provided:
- *Deeper understanding* and a
- *Better flow of argument*.

When explaining their preferred modality for online activities, the 67 participants provided 78 reasons, rather fewer than the 102 that they had given about face-to-face classes.

- *Convenience***:** The most common theme was a practical one; the greater convenience of being able to complete the online activities in their own time (within the given week), and/or at any location, as in the comment, *Can do it at anytime, and anywhere.*
- *Wider contributions:* The second most common theme was that the online discussion allowed contributions from a greater range of people than a class discussion, in which shyer students sometimes stayed quiet in the presence of their more outspoken peers, for example, *Discussion not dominated by loud, confident people.*
- *More detailed responses:* Students also noted that the online discussion forum encouraged more detailed responses than in-class discussion (where conversational turns were typically shorter, but more frequent, than in writing), as in the response, *I thought it was good to be able to read everyone's experiences with full details, as time restraints in class don't allow for each individual to thoroughly go through their task.*

Together, 84% of first reasons (and 69% of second reasons) fitted these three themes. The remaining responses were categorized into smaller themes; specifically:

- *More time to think*: Some students noted that providing responses online gave them more time to consider their answers than having to speak spontaneously in class.
- *Faster to type*: It was noted that it was faster to provide answers to the test when it was typed online than handwritten in the classroom.
- *Less judgment*: Finally, some students felt less judged by their tutor and peers when answers could be written than spoken.

Overall, many of the reasons that students gave for each modality differed from those seen in previous literature, as is taken up again in the General Discussion.

## Study 2

In Study 1, students had been asked to reflect on their preferences and perceptions of several different activities simultaneously: written exercises, class discussion, and a short test. However, the study was not set up to allow students to rate whether they would prefer to do some of these activities in one modality and others in another (these preferences emerged only in the qualitative comments). Further, participants also had to come up with “one good thing” about both face-to-face and online learning, even though some responses suggested that students had simply felt obliged to come up with benefits that they did not really see as an advantage, for example, *[Online activity] can be done in the students own time… but since there is a dedicated time each week for a practical class, this kind of negates this advantage*.

In order to assess students' ratings of the activities separately, and to ask them only for the reasons why they preferred one modality to another, we conducted a second study. In this follow-up, we asked a new group of undergraduates to reflect on their preferences for doing two separate activity types face-to-face or online: short written exercises to get students thinking about the topic (which would traditionally have been done in class but could just as well be done alone, in students' own time), and class discussions.

### Methods

#### Participants

Participants were 37 third-year undergraduate psychology students (10 male) at the same Australian university, who had not participated in Study 1. Their mean age was 25 years (*SD* 8.9 years). All but one spoke English as their first language, and they participated as part of their course requirements. As in Study 1, all were competent and familiar with the technical requirements of the online learning activities. Ethics approval was provided by the university's Human Research Ethics Committee, and participants gave consent for their data to be included in the research.

#### Materials and Procedure

Participants were completing an undergraduate unit which included both face-to-face and online practical classes, and so they were accustomed to both modalities. In this particular unit (Psychology of Language), students had participated in each type of practical class in the last 2 weeks. In both the first week (class conducted face-to-face) and the second week (class conducted online), students had completed written activities, and participated in a discussion, and so the experience of both should have been relatively fresh in their minds. Specifically, in the second week's online practical class, students had completed written activities on language and gender (writing down the best word to address unknown people of varying age/sex, e.g., *sir*, *mate*, *miss*), language in the digital age (responding to fictitious text messages), and language as a marketing tool (comparing two university courses on the basis of the universities' self-descriptions). That same week, students then contributed to an online discussion about their responses to these tasks. The following week, in a face-to-face class, participants discussed their responses to the previous week's tasks. They were then asked to take a few minutes to consider the advantages and disadvantages of completing the written activities online, rather than in a face-to-face class. Even though the students had not done these particular written activities in class, only online, we considered that they would be easily able to make this judgment, because they had previous experience with doing similar kinds of written activities in class on multiple occasions. Students were also asked to consider the advantages and disadvantages of engaging in discussion of these particular language-based issues in class, and online (both of which they had experienced).

Finally, students were asked to:

1. Rate, on a scale of 1 (“much prefer to do in class”) to 5 (“much prefer to do online”), how they would prefer to complete these *written activities*, and to write down their main reason for this rating.
2. Rate, on the same scale, how they would prefer to engage in this *class discussion*, and to write down their meaning reason for this rating.

### Results and discussion

#### Preferences

Overall, participants showed a mean preference for written activities that was close to the middle of the 5-point scale, 3.54 (*SD* = 1.37). In contrast, their mean preference for discussions was closer to the “much prefer to do in class” end of the scale, 1.62 (*SD* = 1.01). The difference between the two ratings was significant, *F*_(1, 36)_ = 59.3, *p* < 0.001. It might be expected that some students simply preferred to do all activities in class or online, but this did not appear to be the case: there was no significant correlation between modality preferences for written activities and discussion, *r* = 0.21, *p* = 0.21. Nor was there any significant correlation with age and either of the modality preferences, written activities *r* = 0.17, *p* = 0.31 or discussion, *r* = 0.06, *p* = 0.71. Thus, it was clear that students had different preferences according to the nature of the task, preferring to do written activities online but discussions face-to-face.

#### Qualitative comments

We also asked participants to note the main reason for their ratings. Using the same process as in Study 1, we conducted a thematic analysis on the responses. Again, some participants provided two reasons, and we coded both of these.

Table 3 shows the themes which came out of the responses about preferences for written activities. As shown in the top panel, only 9 participants (much) preferred doing the written activities in class, while 21 (much) preferred doing the written activities online, as shown in the bottom panel. (The responses from the 5 participants who didn't mind either way were omitted.) As in Study 1, we conducted a chi-square analysis, and confirmed that significantly more participants preferred to do written activities online (or did not mind either way) than to do them in class, χ^2^_(1)_ = 8.26, *p* < 0.005.

**Table 3:** **Study 2: Proportion of Responses per Theme for Written Activities, for those who Preferred these Face-to-Face vs. Online**.

**FACE-TO-FACE**					
**Theme**	**Interaction encourages learning**	**Class time already allocated**	**Immediate feedback**	**Deeper learning**	
Reason 1 (*n* = 9)	0.44	0.33	0.22	0	
Reason 2 (*n* = 7)	0.43	0.14	0.14	0.29	
**ONLINE**					
**Theme**	**More convenient**	**More time to think**	**Right level for online**	**Would waste time in class**	**Less distraction online**
Reason 1 (*n* = 21)	0.71	0.10	0.10	0.10	0
Reason 2 (*n* = 3)	0.66	0	0	0	0.33

The themes emerging from the responses of the relatively small group of participants who preferred doing written activities in class were:

- *Interaction encourages learning*: The students noted that the interaction of face-to-face classes encouraged learning, in a way that could not be achieved via the solitary completion of the activities, for example, *Because if feels like more active learning in class, interacting and talking with others.*
- *Class time already allocated*: A second theme was that since the class time was already allocated for doing activities, students would rather do the work in class, with others, rather than have to force themselves to do it in their own time, as in, *Because I find it hard to make time for non-attending pracs and when I do I rush through them as fast as I can. Whereas if I have to come to class I'm more likely to concentrate and understand what I'm doing.*

Finally, some students valued the

- *Immediate feedback* offered in response to any comments they made
- *Deeper learning* that occurred in class, where they felt more focused on the activities.

The larger group of participants who preferred doing written activities online overwhelmingly reported the following theme:

- *Convenience*: Students noted the convenience of being able to complete the work at a time and place that suited them, as in, *If its online I can do it in bed*.

Other responses fitted the themes of finding that doing the activities online provided:

- More time to think
- Less distraction

Finally, some students noted that it depended on the nature of the written activities—but when they were reasonably challenging but not really difficult (as the current activities were perceived), they were the

- *Right level* for doing online, and
- *Would waste time* if done in class as individual work.

The themes which emerged from participants' preferences about class discussions are summarized in Table 4. Almost all participants (much) preferred discussions in class (*n* = 26, top panel), with very few (much) preferring them online (*n* = 3). (The 6 participants who didn't mind either way were not included in this analysis.) A chi-square analysis confirmed that significantly more students preferred to engage in discussions in the classroom than online (or did not mind either way), χ^2^_(1)_ = 8.26, *p* < 0.005. There was a range of reasons for preferring in-class discussion, with the following themes:

- *More engagement:* The most common theme to emerge was that it allowed more engagement than online discussion, for example, *I would rather be in a classroom talking to actual people and engaging more.*
- *Better flow of discussion:* Another popular theme was that discussion flowed better in person than online, as in, *Can actually have a free flowing conversation*.
- *Personal setting:* Some participants noted that the personal setting of the classroom encouraged better discussion than the more impersonal online environment, for example, *The discussion in person is more beneficial for learning. Easier to communicate and express ideas in a personal setting*.
- *Greater range of opinions:* Students expressed that in-class discussion exposed them to more opinions, for example, *Get more opinions and discussion of them in class*.

**Table 4:** **Study 2: Proportion of Responses per Theme for Discussion, for those who Preferred Discussion Face-to-Face vs. Online**.

**FACE-TO-FACE**								
**Theme**	**More engagement**	**Better flow of discussion**	**Personal setting**	**Greater range of opinions**	**No wish to read others' comments**	**Feedback**	**Deeper learning**	**Time already allocated**
Reason 1 (*n* = 26)	0.31	0.23	0.15	0.12	0.08	0.08	0	0.04
Reason 2 (*n* = 17)	0	0.12	0.18	0.24	0.24	0.06	0.18	0
**ONLINE**								
**Theme**	**More time to think**	**Wastes class time**	**Deeper learning**					
Reason 1 (*n* = 3)	0.33	0.33	0.33					

The remaining comments expressed the themes of:

- *No wish to read others' comments* (although they were happy to listen to them)
- *Immediate feedback* of face-to-face discussion
- *Deeper learning* facilitated by in-class discussion
- *Time already allocated* in class, so students were happy to do the discussion then.

As shown in Table 4, only three participants said that they preferred discussions online, each with a separate reason.

## General discussion

The goal of this research was to explore whether students' academic marks and their preferences for learning varied as a function of online or offline delivery. A further aim was to illuminate the “active ingredients” contributing to students' experiences in these modalities, using a qualitative methodology. In two studies, undergraduate students engaged in practical class activities in two modalities: in a face-to-face class, and online. We assessed students' academic performance on tests of class content and asked students which modality they preferred for learning, and why. Overall, we saw no clear differences in academic performance in online vs. in-class learning, but students had a general preference for in-class activities, specifically when discussion of academic topics was required.

In terms of academic performance, our first study showed that there were no significant differences in test performance whether class material, and the subsequent test, was presented face-to-face or online. This finding adds to previous evidence that simply participating in online activities does not necessarily lead to significantly improved test scores (Davies and Graff, 2005). On the surface, these findings are difficult to explain using student-centered models of e-learning: it is assumed that the self-directed learning that occurs in in online environments should result in deeper learning. However, an alternative explanation comes from Garrison (2012), who notes that the community of inquiry created by e-learning is not synonymous with the collaborative and constructivist approaches that can foster deep learning. If so, perhaps the current findings reflect collaboration and constructivism in both modalities, but in regards to different aspects of the learner experience. Overall, performance across the two modalities is convergent, but the electronic and face-to-face pathways to that performance may be divergent. For example, the benefits obtained a student who has “more time to think” when working asynchronously online may be similar to the benefits obtained by another student “exposed to more opinions” within a classroom discussion. Future research could aim to unpack such benefits and their relationship with student characteristics: this could potentially results in truly student-centered approaches through the creation of bespoke learning activities.

There was also no significant link between the academic mark achieved on the unit overall and preference for either face-to-face or online learning activities. The lack of relationship between preference and performance is a notable one. At least in the current study, it seems that a liking for a particular modality does not benefit performance in that modality. Importantly, these findings also suggest that asking students to engage in their non-preferred modality does not mean that poor performance will ensue. The implication is that educators can select delivery modality based solely on pedagogical reasons with confidence, rather than being concerned that a certain delivery method may disadvantage students who hold negative perceptions of that method.

The participants in our first study preferred, on average, to complete activities in class rather than online. In the second study, participants had the opportunity to be more specific about their preferences. Their responses indicated that although they were happy to complete individual written exercises online (especially if these were of a reasonable level of difficulty), students much preferred to participate in discussions in face-to-face classes. Thematic analysis (Braun and Clarke, 2006) of participants' qualitative comments in both Studies 1 and 2 revealed that students most liked the greater sense of engagement provided by face-to-face vs. online activities. Since there appears to be no previous direct comparison of students' perceptions of learning in these two modalities, it is difficult to link these responses with those in the literature concerning only online learning. However, it is interesting to note that the most common theme emerging in both studies was about the stronger feeling of engagement in the subject matter that these activities provided, with many students noting the deeper learning to come from this greater engagement. Although this result in no way negates previous findings that e-learning can also encourage deep learning (e.g., Ramsden, 1992; Robinson and Hullinger, 2008), it should be borne in mind that the benefits of e-learning do not diminish any pre-existing benefits of traditional classroom learning.

In Study 1, the immediate feedback from peers and tutor was also noted as an important advantage of in-class activities in general. In contrast, the immediacy of feedback available from online activities can vary greatly, depending on the nature of the activities and the way a unit is run. In the current study, students had 5 days in which to conduct their on-line discussion with peers. Thus, some students had to wait hours or even days for a peer to comment on their particular contribution, a situation that could not arise in an in-class discussion. In other units or courses, online discussions could be organized to occur at a set time, or as a “chat” function, in real time. This would allow contributions to be responded to more rapidly, and would presumably alleviate some of the frustrations that our participants experienced with more drawn-out discussions. In Study 2, in addition to preferring in-class discussion because of the greater engagement it offered, students also expressed the themes that face-to-face discussion allowed better flow of conversation, provided a more personal setting in which to enjoy the discussion, and encouraged a greater range of opinions from the group. These findings highlight the importance of facilitating collaboration and constructivism in learning.

When students were asked to note “one good thing” about online learning (Study 1), or to explain why they preferred to do written exercises online (the majority of participants in Study 2), thematic analysis revealed an important common theme: convenience. Students in both studies noted for online learning; they could do the work at a time of their own choosing, without having to travel to the university. This pragmatic reason emphasizes the advantages of online activities for many students, but does not fit with the more pedagogically driven reasons seen in previous research (e.g., Galway et al., 2014). Nevertheless, it does suggest that at least some students appreciate the flexibility in time and location afforded by online activities, especially as part of an otherwise face-to-face unit.

A number of participants in the first study also noted that online work encouraged contributions from a wider range of students, specifically, those who might be shy about face-to-face interaction. This reason has been noted in previous work (e.g., Citera, 1998; Hobbs, 2002). Nevertheless, on its own, this point does not provide a strong justification to adopt online learning, as many universities now consider oral communication skills as an important graduate attribute (e.g., Australian Qualifications Framework, 2013). Only a few students provided an academically oriented benefit of online learning, noting that it encouraged more detailed answers, and that contributors had more time to think before responding, compared to in a face-to-face class. These reasons thus seemed to be less important to our participants than to participants in other studies (e.g., Robinson and Hullinger, 2008).

Another finding to emerge from Study 1 was that students' preference for one modality over the other was not related to their marks on the related tests, nor to their age. Much of the extant research on online learning has focused on students who might begin at a disadvantage because they do not have the skills to interact confidently with the technology (e.g., Zhang and Perris, 2004). Further, as noted by Ituma (2011), it seems that when students do not struggle with the technical requirements of online learning, age is no barrier to the value that they can gain from this modality of study. However, with the increased use of e-learning in mainstream education, online components are part of the university experience for more and more students from a diverse range of backgrounds (e.g., Chen et al., 2010; Holley and Oliver, 2010), and appropriate technological skills should not be assumed.

The present results serve as a reminder that it is rather simplistic to consider “online learning” as a unitary concept, to be examined on its own or compared wholesale to face-to-face learning. Both modalities of education have multiple aspects, and research focusing on one particular aspect or combination of aspects might reach very different conclusions from research focusing on another aspect or combination of aspects. Our participants liked completing individual written activities online, but preferred to engage in class discussions in person. As noted earlier, this view may have been strongly influenced by the stilted and drawn-out nature of the online discussion that the current participants experienced, which took place mostly asynchronously, rather than in real time. It is clear that the developers of online content need to consider a range of issues in designing the best way to deliver this content to students. Simply providing materials (Evans et al., 2004) or a discussion forum (Swan, 2002) online does not automatically aid learning (Davies and Graff, 2005), making more contributions to an online discussion does not necessarily lead to better academic performance.

In addition to some of the limitations already mentioned, it should be noted that our sample sizes were modest. The participants were all advanced level undergraduates in psychology, who were enrolled in a face-to-face course but who were also accustomed to doing some of their learning online, in a self-directed way. Thus, further work with a wider range of participants would help to ascertain the generalizability of these findings. It should also be noted that we asked students about only a few aspects of online vs. face-to-face learning, in a unit which provided online materials in a relatively limited range of ways (e.g., asynchronous discussion forum, scrollable written exercises). This specificity is both a strength and a limitation of the current research. Although they provide good experimental control and strong ecological validity, the focused nature of these tasks and their context means that it is important not to overgeneralize our findings. Future research should aim to extend questions about online learning to a wider range of disciplines, using online activities in a more varied range of ways, in order to build up a broader picture of students' preferences and performance in terms of online and in-class learning. Extending research in a number of dimensions will become increasingly important for understanding how blended learning and flipped classrooms can progress along with the technology that underpins these paradigms.

The provision of online units, and of online components to face-to-face units, is continuing to expand worldwide. Those who are responsible for creating online material for blended units would do well to consider carefully the nature and type of activities they allocate to each modality. In directly comparing the same students' performance and perceptions on in-class vs. online learning, this study confirmed that in these groups, at least, online activities led to similar levels of academic achievement as face-to-face activities. It seems that although students appreciate the flexibility of choosing the time and place to do some activities, they also value the greater engagement provided by discussions that take place face-to-face, rather than face-to-screen. Rather than being seen simply as an alternative modality for delivering academic content, the benefits of online technology should be adapted not only to offer greater flexibility, but to inspire students' engagement and success at university and beyond.

### Conflict of interest statement

The authors declare that the research was conducted in the absence of any commercial or financial relationships that could be construed as a potential conflict of interest.
